# Long-Term Survival Outcomes and Treatment Experience of 64 Patients With Esthesioneuroblastoma

**DOI:** 10.3389/fonc.2021.624960

**Published:** 2021-03-04

**Authors:** Qian Zeng, Yifu Tian, Yihong He, Qiongxuan Xie, Ludi Ou, Min Wang, Wen Chen, Rui Wei

**Affiliations:** ^1^Department of Oncology, Xiangya Hospital, Central South University, Changsha, China; ^2^National Clinical Research Center for Geriatric Disorders, Xiangya Hospital, Central South University, Changsha, China; ^3^Department of Pathology, Xiangya Hospital, Central South University, Changsha, China

**Keywords:** esthesioneuroblastoma, prognostic factors, treatment, survival, radiotherapy, endoscopic surgery

## Abstract

**Background:** Esthesioneuroblastoma (ENB) is a rare sinonasal malignancy, lacking a unified staging system and treatment. Management at a single center was retrospectively evaluated to inform future treatment options and prognostic factors.

**Methods:** Clinical data of 64 consecutive ENB patients, including prognostic factors and treatment methods, were reviewed retrospectively. Data were collected to calculate overall survival (OS) and progression free survival (PFS).

**Results:** The majority of tumors 84.4% were within Kadish C stage, 79.7% were within T3 or T4, and 64.0% were within Hyams grade III or IV. A total of 50 (78.1%) patients received surgery and combined radiotherapy with or without chemotherapy, 10 (15.6%) received surgery with or without chemotherapy alone, and 4 (6.3%) received radiotherapy with or without chemotherapy alone. The majority of patients (79.7%) underwent endoscopic resection (endoscopic and endoscopically assisted). Surgery combined with radiotherapy with or without chemotherapy resulted in significantly better OS (84.4 vs. 50.6%, 84.4 vs. 37.5%) compared to surgery alone and radiotherapy alone (*P* = 0.0064). Endoscopic surgery group (endoscopic and endoscopically assisted) resulted in significantly better 5-year PFS (61.7 vs. 22.2%) compared to the open surgery group (*P* < 0.001). Although endoscopic surgery group was not a statistically significant predictor of 5-year OS (*P* = 0.54), the 5-year OS was 79.3% for the endoscopic surgery group and 76.2% for the open surgery group. A Cox regression analysis identified intracranial extension and surgery combined with radiotherapy as independent factors affecting 5-year OS while cervical lymph node metastasis and Hyams grade IV as independent factors affecting 5-year PFS.

**Conclusion:** Our findings suggest that surgery combined with radiotherapy is the best treatment approach for ENB. For advanced tumors, endoscopic surgery is an effective treatment, and its survival rate is equal to or better than open surgery.

## Introduction

Esthesioneuroblastoma (ENB), also called olfactory neuroblastoma, is a primitive neuroectodermal tumor which is believed to originate from the olfactory epithelium of the upper nasal cavity. It was first described by Berger et al. in 1924 and named “esthesioneuroepitheliome olfactif.” ENB is characterized by a prolonged natural history (1) and varying biological activity, ranging from a less invasive tumor leading to long-term survival, to a highly invasive neoplasm leading to rapid extensive metastasis with limited survival.

The clinical manifestations of ENB are non-specific. The main clinical manifestations are nasal obstruction and epistaxis. ENB is easily misdiagnosed as other tumors, such as small cell carcinoma, melanoma, rhabdomyosarcoma, pituitary adenoma, sinonasal undifferentiated carcinoma, sinonasal neuroendocrine carcinoma, and lymphoma (2). A combination of physical examination, nasal endoscopy, imaging examinations, and pathology are required to confirm the diagnosis. Pathology is the gold standard for the diagnosis of ENB. The pathological features of ENB include uniform small round cells, eosinophilic fiber background, true rosette (Flexner-Wintersteiner rosettes), or pseudorosette (Homer Wright rosettes) formations. The typical immunohistochemical positive markers of ENB are neuron-specific enolase, S-100, chromogranin A, and synaptophysin.

The treatment of ENB can be complex due to its infrequent occurrence, special anatomy, a lack of consistent treatment, no uniform staging system, and few prospective trials. Surgical resection has been the mainstay of treatment, and additional radiation therapy is usually used for advanced tumors. This is because in more advanced tumors, it is often difficult to obtain a clear margin. Surgery or radiotherapy alone is recommended in stage A patients (3–5); however, in select stage A patients, including those who have not obtained a negative margin or who have an invasive tumor, it is necessary to opt for surgery combined with radiotherapy (6). Surgery combined with radiotherapy is regarded as the gold standard by most institutions (7–9).

There is no universally accepted staging system for ENB. In 1976, Kadish et al. (10) developed a classification system based on local extension which was then modified by Morita et al. (11) in 1993. A grading system based on pathological features was developed by Hyams (12) in 1982, while a TNM-based classification system was created in in 1992 by Dulguerov and Calcaterra (13). The Kadish staging system, however, remains the most commonly used staging system and has been used as an independent predictor of outcome (14). Previous studies have also found the Hyams grading system to be a useful predictor of survival and capable of guiding appropriate treatment (14, 15). However, some researchers doubt these results as they were derived from a small sample, single-center study. Further evaluation of the value of the staging systems in ENB is therefore warranted.

We collected information from 64 ENB patients at our institution between 2000 and 2018. Here, we aimed to investigate the basic characteristics of these patients, treatments, underlying prognostic factors of the disease, and long-term survival outcomes. We further sought to identify the best surgical approach for ENB and to introduce the application of modern radiotherapy technology in ENB.

### Patient Data

A total of 64 patients admitted to Xiangya Hospital, Central South University between March 2000 and December 2018 were identified; Records from all patients were sufficiently comprehensive for analysis in this study. Patients with a history of malignancy or a second primary tumor were excluded. The median follow-up for our cohort was 48.1 months (range, 1–226 months). Follow- up visits included nasal endoscopy and magnetic resonance imaging. During the follow-up, the patients were examined every 1–3 months in the first year, every 2–4 months in the second year, and every 4–6 months in the third to five years in our study. This retrospective study was approved by the ethics committee of our hospital. We collected medical records, including demographic information, initial diagnosis, tumor staging, treatment modalities, and survival outcomes.

### Treatment Modalities

The management of ENB is controversial and variable. Patients diagnosed with ENB received surgical resection, radiotherapy, and chemotherapy, or a combination of these methods. Of the total 64 patients, 50 (78.1%) received surgery and combined radiotherapy with or without chemotherapy, 10 (15.6%) received surgery with or without chemotherapy alone, and 4 (6.3%) received radiotherapy with or without chemotherapy because they refused surgery or the tumor burden was too large to perform surgery. The surgical approaches mainly included open craniofacial resection (CFR) and endoscopic surgery (endoscopic and endoscopically assisted). The approach was open CFR in 14.0% (*n* = 9) patients, endoscopic-only in 62.5% (*n* = 40) patients, and endoscopic-assisted in 17.2% (*n* = 11) patients. The majority of patients (79.7%) underwent endoscopic resection, with 65.6% being Kadish C stage and 74.5% being T3 or T4. Chemotherapy was carried out in the induction, concurrent and adjuvant settings, with variable regimens. Chemotherapy was delivered in 30 patients, while chemotherapy status in 10 patients was unknown in our series. The regimens used for chemotherapy included etoposide and cisplatin, and docetaxel and cisplatin in the majority of patients. Adriamycin, cyclophosphamide and vincristine were also used in some patients. A total of 50 patients were irradiated with intensity-modulated radiotherapy (IMRT) or Hi-Art helical tomotherapy (HT). Each patient was immobilized with a custom-made thermoplastic cast in the supine position. All subjects were scanned with 3 mm slice thickness using a Siemens Plus 4 Spiral computed tomography (CT) simulator. Scanning started from the top of the head to the bifurcation of trachea, with MRI scanning in the same fixed position. HT plans were drawn up on Tomotherapy Hi-Art Software (version 2.0.7) (Accuray, Madison, WI, USA), while IMRT plans were generated by Eclipse TPS (Varian Medical Systems Inc., version 11.0.31). All gross tumor volume (GTV) images (consisting of tumor bed and residual) were contoured by the same radiologist and confirmed by an experienced radiation oncologist. The clinical target volume (CTV) was defined as the grossly detectable tumor volume plus microscopic tumors, which included low-risk CTV and high-risk CTV. A planned target volume (PTV) with a margin of 3 mm was usually added to the CTV. The treatment was administered 5 days per week with a single dose of 2–2.24 Gy for all patients. Radiation dose ranged from 54–75.9 Gy (median 66.2 Gy) with IMRT as post-operative radiotherapy. Radiation dose ranged from 69.4–72.8 Gy (median 71.1 Gy) with IMRT as definitive radiotherapy. Only 1 patient was treated with HT as the post-operative radiotherapy: the radiation dose was 60 Gy, and the single dose was 2 Gy. A total of 7 patients had lymph node metastasis, and 2 of them received adjuvant irradiation of the neck with a median dose of 64.95 Gy. A total of 8 patients received prophylactic neck radiation therapy, and the total dose ranged from 65.7 to 70.6 Gy (median 69.0 Gy). The following organs were delineated as organs at risk: the lens, optic nerves, optic chiasm, major lacrimal glands, brainstem, spinal cord, temporal lobe, parotid glands, and mandible. A 5, 1, and 5 mm margin was added to the lens, brainstem, and spinal cord, respectively, to create their planning organ at risk volumes (PRVs). The different treatments are presented in Table 1.

**Table 1:** Treatments for patients with esthesioneuroblastoma.

**Treatments**	**Total patients**	**%**
**Surgery**		
Open surgery	9	14.0
Endoscopic	40	62.5
Endoscopic assisted	11	17.2
No surgery	4	6.3
**Radiation treatment**		
None	10	15.6
Post-operative	48	75.0
Pre-operative	2	3.1
Definitive	4	6.3
**Chemotherapy**		
No	24	37.5
Yes	30	46.9
Induction	3	
Induction and concurrent	1	
Induction + concurrent + Adjuvant	1	
Concurrent	5	
Concurrent and Adjuvant	1	
Adjuvant	19	
Unclear	10	15.6

### Statistical Analyses

The primary endpoints for our study were overall survival (OS), progression-free survival (PFS), locoregional relapse-free survival (LRFS), and distant metastasis-free survival (DMFS). Survival was calculated from the start of primary treatment to the date of death, progression, recurrence of the primary site or cervical lymph nodal involvement, and distant metastasis. All survival outcomes were evaluated using the Kaplan–Meier method and compared using the log-rank test. The Cox proportional hazards model was used to identify independent prognostic factors for OS and PFS. All data analyses were performed using IBM SPSS software (version 20.0; Chicago, IL, USA), with *P* < 0.05 considered statistically significant.

## Results

### Patients' Characteristics

Among all included patients, 40 patients were male and 24 patients were female. Age at presentation ranged from 16 to 79 years, (mean, 47.0 years). The age of onset of ENB did not reveal a bimodal distribution. The presenting symptoms were nasal obstruction (*n* = 33), epistaxis (*n* = 30), headache (*n* = 27), anosmia (*n* = 21), vision loss (*n* = 8), purulent mucus (*n* = 6), epiphora (*n* = 4), neck mass (*n* = 2), exopthalmos (*n* = 1), and disturbance of consciousness (*n* = 1). The average period from symptom onset to diagnosis was 14 months (range 0.5–240 months). The basic clinical data of patients is presented in Table 2. A total of 20 cases had orbital involvement, and there was brain involvement in 26 cases.

**Table 2:** Characteristics for patients with esthesioneuroblastoma.

**Characteristics**	**Total patients**	**Range or %**
**Age**		
Range		16–79 y
<50 y	35	54.7
>50 y	29	45.3
**Sex**		
Male	40	62.5
Female	24	37.5
**Staging**		
**T classification**		
T1 or T2	13	20.3
T3	25	39.1
T4	26	40.6
**Kadish stage**		
A or B	10	15.6
C	54	84.4
**Hamys grade**		
1 or 2	23	36.0
3	26	40.6
4	15	23.4
**Lymph node metastasis**		
(+)	7	10.9
(–)	57	89.1
**Distant metastasis**		
(+)	3	4.7
(–)	61	95.3
Orbital invasion	31	48.4
Intracranial extension	26	40.6

Dulguerov (13), Kadish (10), and Hyams (12) classifications were reported. Thirteen (20.3%), 25 (39.1%), and 26 (40.6%) patients had T1 or T2, T3, and T4 respectively; A total of 10 (15.6%) and 54 (84.4%) patients had Kadish stage A or B, and C, respectively; 23 patients (36.0%) had Hyams grade I or II, 26 patients (40.6%) had Hyams grade III, and 15 patients (23.4%) had Hyams grade IV. Hyams I–II and III–IV were categorized as low-grade Hyams or high-grade Hyams, respectively.

### Survival in Association With Treatment Modality

Of the 64 patients, 13 died during follow-up. The 5-year OS, PFS, LRFS, and DMFS rates of our cohort were 76.5, 54.7, 68.7, and 87.7%, respectively. The 5-year OS rates for surgery combined with radiotherapy, surgery alone, and radiation alone with or without chemotherapy were 84.4, 50.6, and 37.5% (*P* = 0.0064), respectively. The 5-year PFS rates for surgery combined with radiotherapy, surgery alone, and radiation alone with or without chemotherapy were 60.7, 30.5, and 37.5% (*P* = 0.11), respectively. The 5-year OS rates for the radiation group and no-radiation group were 81.3 and 50.6% (*P* = 0.042), respectively. The 5-year PFS rates for the radiation group and no-radiation group were 59.3 and 30.5% (*P* = 0.073), respectively. The 5-year OS rates for the surgical and non-surgical group were 78.8 and 37.5% (*P* = 0.024), respectively. The 5-year PFS rates for the surgical and non-surgical group were 55.7 and 37.5% (*P* = 0.35), respectively. The 5-year OS rate for the endoscopic surgery group (endoscopic and endoscopic-assisted), and open surgery group were 79.3 and 76.2% (*P* = 0.54). The 5-year PFS rate for the endoscopic surgery group (endoscopic and endoscopic-assisted), and open surgery group were 61.7 and 22.2% (*P* < 0.001).

### Survival in Association With Staging System

According to the TNM staging, the 5-year OS rates for T1 or T2, T3, and T4 were 100, 77.9, and 65.4% (*P* = 0.0075), respectively. The 5-year PFS rates for T1 or T2, T3, and T4 were 73.8, 52.7, and 49.3% (*P* = 0.14), respectively. According to Kadish stage, the 5-year OS rates for stages A or B, and C were 100 and 70.8% (*P* = 0.079), respectively. The 5-year PFS rates for stages A or B, and C were 90 and 45.8% (*P* = 0.035), respectively. According to the Hyams grade, the 5-year OS rates for grade I or II, III, and IV were 89.8, 73.7, and 57.8% (*P* = 0.015), respectively. The 5-year PFS rates for grade I or II, III, and IV were 77.7, 47.6, and 33.3% (*P* = 0.0019), respectively.

### Survival in Association With Various Clinicopathological Factors

Patients with and without cervical lymph node metastasis showed differences in OS (53.6 vs. 80.2%, *P* = 0.29) and PFS (14.3 vs. 62.9%, *P* = 0.00026). Patients with and without distant metastasis showed differences in OS (66.7 vs.76.8%, *P* = 0.93) and PFS (33.3 vs. 57.9%, *P* = 0.018). Patients with and without intracranial extension showed differences in OS (65.4 vs. 86.0%, *P* = 0.004) and PFS (49.3 vs.60.5%, *P* = 0.075). Patients with and without orbital involvement showed differences in OS (69.2 vs.79.8%, *P* = 0.36) and PFS (57.9 vs. 53.3%, *P* = 0.69).

### Prognostic Analysis

**Figure 1:**
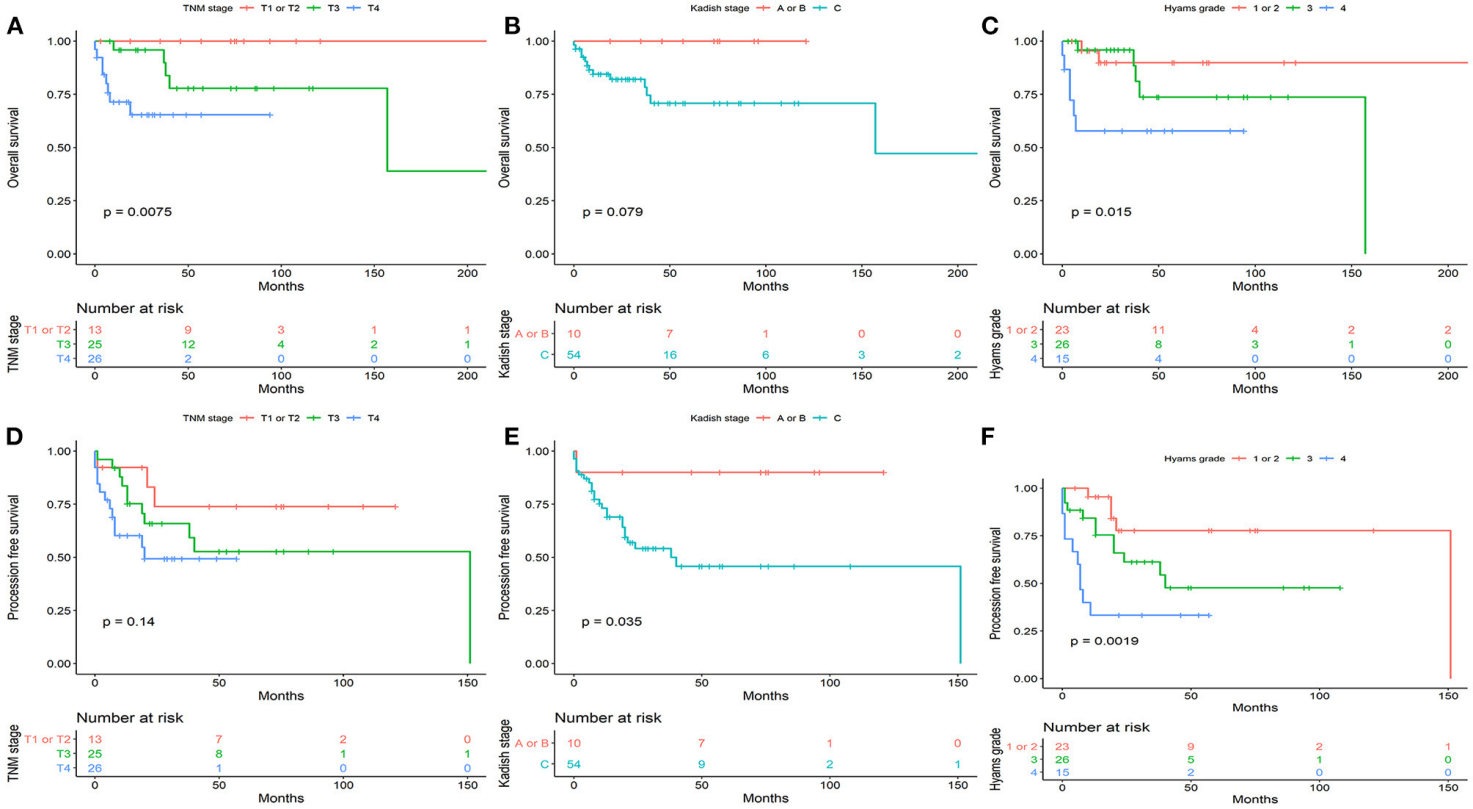
The overall survival **(A–C)** and progression -free survival **(D–F)** stratified by TNM-based classification system, Kadish stage, and Hyams grade.

**Figure 2:**
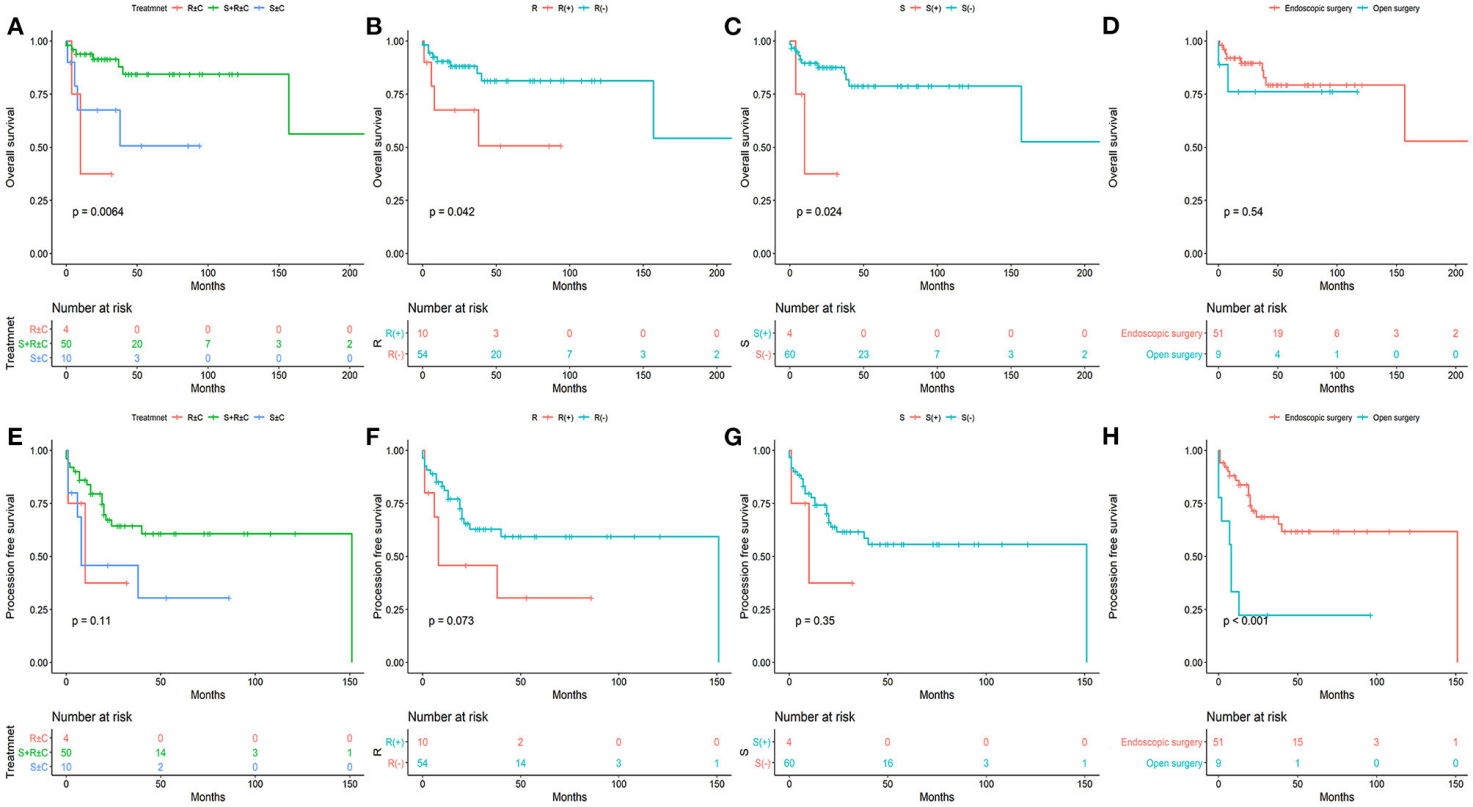
The overall survival **(A–D)** and progression -free survival **(E–H)** stratified by different treatments.

**Figure 3:**
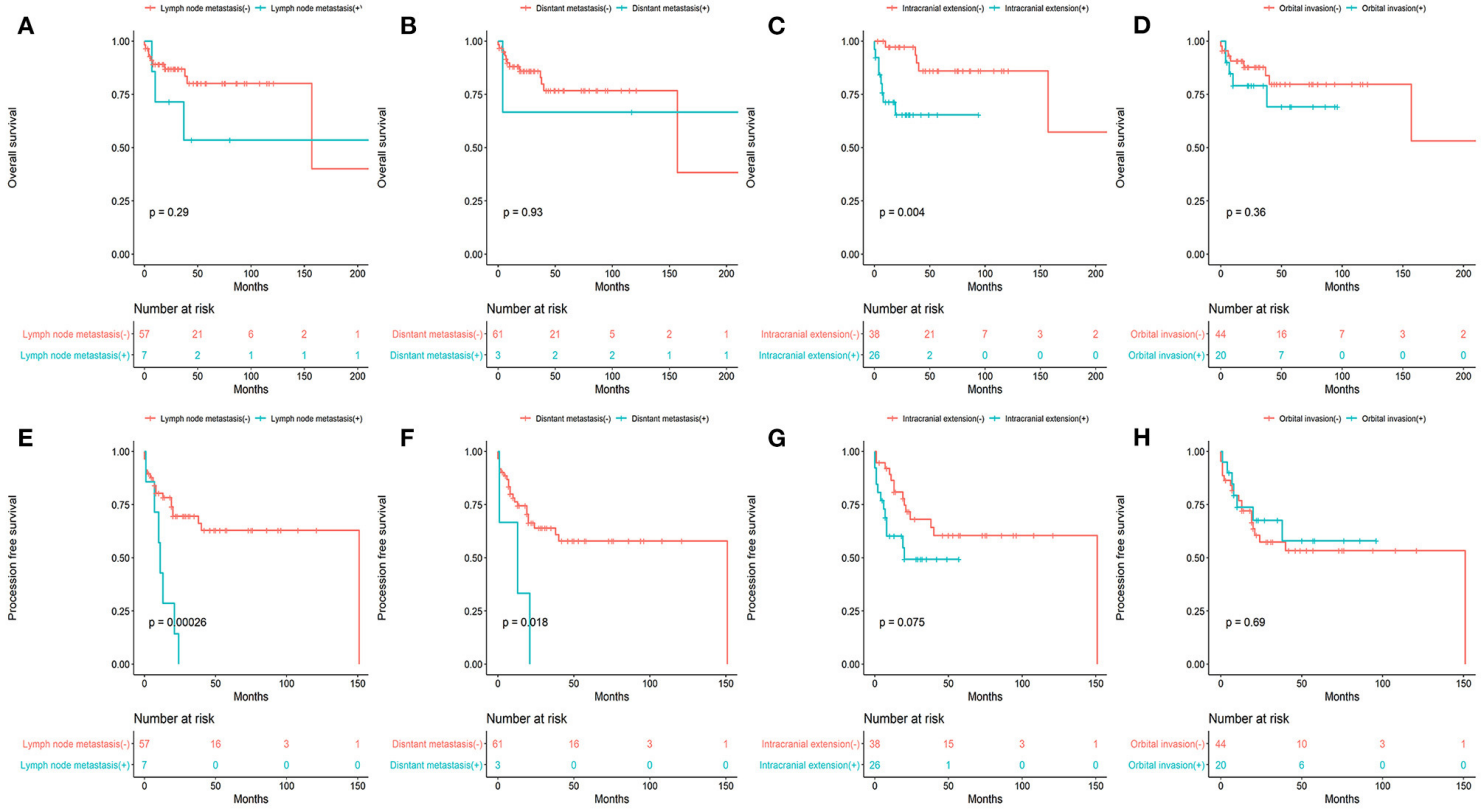
The overall survival **(A–D)** and progression -free survival **(E–H)** stratified by various clinicopathologic factors.

The results of univariate analysis are presented in Figures 1–3. Multivariate analysis identified intracranial extension as an independent prognostic factor for poor OS [hazard ratio (HR) = 4.72, 95% confidence interval, (CI) = 1.372–16.245, *P* = 0.014] and cervical lymph node metastasis as an independent prognostic factor for poor PFS (HR = 5.426, 95% CI= 1.942–15.16, *P* = 0.001). Among the various treatment modalities, primary RT alone was used as a reference, and surgery combined with radiotherapy exhibited a significant improvement in OS (HR = 0.167, 95% CI = 0.032–0.873, *P* = 0.034), while the surgery alone did not (HR = 0.655, 95% CI = 0.113–3.812, *P* = 0.638). Among the Hyams grades, Hyams grade I or II was used as a reference, Hyams grade IV exhibited a significant poor PFS (HR =4.363, 95% CI = 1.118–17.026, *P* = 0.034), while Hyams grade III did not (HR = 2.196, 95% CI = 0.648–7.442, *P* = 0.207).

### Failure Patterns and Treatments

Of the 64 patients, local recurrences were found in 14 (21.9%) patients, with a median time to local recurrence of 19 months (range, 0–151). In our cohort, 2 patients presented with cervical lymph node metastasis, and regional relapses occurred in 5 (7.8%) patients during follow-up, with a median time to relapse of 14 months (range, 0–21). Of these patients, 2 out of 7 had bilateral neck lymph node recurrence, with the other 5 patients experiencing ipsilateral recurrence. 5 patients developed level II and III lymph node metastasis, 2 patients developed level IV lymph nodes, and 1 patient developed level I and V. 3 patients developed distant metastasis and 1 patient had combined lymph node recurrence. The most common metastasis sites were the lung (3 patients), paranephros (1 patient), pancreas (1 patient), liver (1 patient), peritoneum (1 patient), pericardial diaphragmatic lymph node (1 patient), and skin (1 patient). When patients relapsed *in situ*, surgery, radiotherapy, chemotherapy, and comprehensive treatment were often selected. Of the 5 patients with cervical lymph node metastasis, 3 patients underwent lymph node dissection and 2 patients underwent lymph node irradiation after lymph node dissection. Of the 2 patients that presented with cervical lymph node metastasis, 1 underwent lymph node dissection, and the other underwent chemotherapy. Of the 7 patients with lymph node metastasis, 3 died. 3 patients with distant metastases were treated with chemotherapy, and 1 patient died 3 months after recurrence and metastasis. The Patterns of recurrence are presented in Table 3.

**Table 3:** Patterns of recurrence.

**Pattern**	**Total patients**	**%**
**Recurred**		
Yes	17	26.6
No	47	73.4
**Site of first recurrence**		
Local	14	21.9
Regional	5	7.8
Distant	3	4.7
**Distant metastasis**		
Yes	3	4.7
No	61	95.3
**Sites of distant metastasis**		
Lung	3	
Liver	1	
Paranephros	1	
Pancreas	1	
Peritoneum	1	
Pericardial diaphragmatic lymph node	1	
Skin	1	

## Discussion

In the present study, the 5-year OS, PFS, LRFS, and DMFS rates were 76.5, 54.7, 68.7, and 87.7%, respectively. The 5-year OS was within reported ranges, and tended to be higher than previously reported outcomes (7, 16, 17). The improvement of surgery and radiotherapy might have also modified patient outcomes. The 5-year PFS and 5-year LRFS rates were worse than a previous 217-patient investigation by Song et al. (18) who reported a 5-year PFS and 5-year LRFS of 79 and 79.3%, respectively. However, their 5-year OS and DMFS rates of 80.0 and 80.0% respectively were comparable to our own. Song et al.'s study represents the largest sample of ENB patients in a single institution. The survival outcomes of ENB in other reports are shown in Table 4. In our study, a high percentage of patients progressed, requiring long-term follow-up and timely salvage treatment.

**Table 4:** Studies reporting survival outcomes for ENB.

**References**	**Year**	**Period**	**Patients**	**Treatment**	**Follow up (mo)**	**OS**	**Other survival rate**
Hwang et al. (16)	2002	1979 −2000	21	Surgery only, Surgery + RT ± CTX, RT ± CTX	28.7	5-Y:21.3%	-
Aboziada et al. (19)	2010	1995–2007	29	Surgery ± CTX, Surgery + Postop RT ± CTX	-	5-Y:100.0%	5-Y:DFS:54.0%
Ow et al. (9)	2014	1992–2007	70	Surgery ± CTX, Surgery + Pre/Postop RT ± CTX,RT ± CTX	91.4	-	-
Mori et al. (20)	2015	1992–2013	17	RT ± CTX, Surgery + Pre/Postop RT ± CTX	95	5-Y:88.0%	5-Y:RFS:74.0%
Rimmer et al. (21)	2014	1978–2013	95	Surgery ± CTX, Surgery + RT ± CTX	88.6	5-Y:83.4%	5-Y:DFS:80.0%
Nakamura et al. (3)	2017	1999–2012	42	RT ± CTX, Surgery + RT ± CTX	69	5-Y:100% Kadish A5-Y:86% Kadish B5-Y:76% Kadish C	5-Y: PFS:80% Kadish A 5-Y: PFS:65% Kadish B 5-Y: PFS:39% Kadish C
Xiong et al. (8)	2017	1981–2015	187	Surgeryonly, Surgery + RT ± CTX, RT ± CTX	34	3-Y:66.7%	3-Y:DFS:57.5%
Yuan et al. (17)	2018	1986–2011	44	Surgery ± CTX, Surgery + Postop RT ± CTX, RT ± CTX	84	5-Y:42.7%	5-Y:PFS:39.1%
Song et al. (18)	2020	1991–2019	217	RT ± CTX, Surgery + Pre/Postop RT ± CTX	58.9	5-Y:80.0%	5-Y: PFS:79.0%
Yin et al. (7)	2016	1979–2914	113	Surgery ± CTX, Surgery + pre /Postop RT ± CTX, RT ± CTX, chemotherapy only	75	5-Y:65.0%	5-Y:LRC:73.0%

*RT, radiotherapy; Postop RT, post-operative radiotherapy; CTX, chemotherapy; DFS, disease-free survival; RFS, relapse-free survival; PFS, progression-free survival; LRC, loco-regional control rate*.

The treatment approaches for ENB still remain controversial, although it is generally believed that a combination of surgery and radiotherapy provides the best results (7–9). However, a meta-analysis of 956 patients showed that the 5-year OS was statistically equivalent (78 vs. 73%) between patients receiving surgery alone and patients receiving comprehensive treatment (14). In our study, surgery combined with radiotherapy with or without chemotherapy resulted in significantly better OS (84.4 vs. 50.6%, 84.4 vs. 37.5%) compared to surgery alone and radiotherapy alone. Because 10 patients lost chemotherapy data, to then analyze the outcome data with a ± chemo could be a serious confounder. In order to explain this problem, sensitivity analysis showed that the effect of chemotherapy was very small, which still supported the original conclusion. The role of chemotherapy is still controversial in ENB, generally used for advanced, high-grade tumors (22–24). However, chemotherapy did not improve OS, RFS or DSS (25, 26). In advanced tumors, we therefore support multimodal therapy. In local early tumors, monotherapy has also been approved. Monotherapy should be considered for tumors of the nasal cavity with a low Hyams grade and less invasiveness.

Surgical resection has been the mainstay of treatment in ENB. With the development of surgical techniques, from open craniofacial resection (CFR) to the development of endoscopic surgery, endoscopic surgery has been welcomed as a treatment for ENB. CFR has become the standard open surgical procedure for ENB before endoscopic surgery. Some reports have demonstrated the efficacy of CFR for ENB (27, 28), as the use of CFR in the treatment of ENB has been shown to improve survival outcomes. No matter what kind of operation, surgeons should abide by the operation principles and indications. When there are diseases in the midpoint of the orbital roof, intraorbital diseases and extensive frontal sinus involvement, CFR is appropriate, but endoscopic surgery is not (29). Endoscopic surgery is usually limited to the nasal cavity and paranasal cavity, but in recent years, the indication of endoscopic surgery is progressively expanding. Previous literatures have well-summarized the indications and contraindications for endoscopic transnasal removal of sinonasal malignancies (30, 31). Endoscopic surgery has been standardized (31), and its safety and feasibility have been confirmed. Shorter hospitalization days, reduced morbidity and mortality rate, higher survival rate, avoidance facial wounds are cited as the major advantages of endoscopic resection compared with CFR (32–34). The incidence of complications after CFR was about 30–60%, and the mortality rate was 0–13% (35). However, the overall complication rate after endoscopic surgery of anterior skull base tumors or sinonasal malignancies was significantly reduced, about 3–29%, and the mortality rate was 0–1% (36, 37).

Endoscopic surgery may be divided into categories: cranioendoscopic approach (CEA), exclusively endoscopic approach (EEA). The act of CEA is more invasive than EEA. Hanna et al. (38) found disease recurrence and survival did not differ significantly between the EEA group and the CEA group, although this observation was not confirmed by Nicolai et al. (39). Whether the invasive behavior of endoscopic surgery will affect survival outcomes needs further study.

In most institutions, CFR is generally chosen for advanced invasive tumors, and nasal endoscopic surgery is usually used for early localized tumors (21, 40). A meta-analysis of 361 patients reported that endoscopic surgery produced a higher survival rate than open surgery for less invasive tumors (41). Suriano et al. reported endoscopic surgery combined with radiotherapy achieved satisfactory results, which can replace open surgery in Kadish stage A and B patients (42). The above research reported that endoscopic surgery and CFR were comparable for early tumors, and endoscopic surgery may produce better survival outcomes. Currently, for invasive tumors, there are few reports comparing the results of open surgery and endoscopic surgery. In patients with Kadish stage C and D or high-grade Hyams, endoscopic surgery demonstrated significantly better survival than open surgery (35). Harvey et al. reported that the endoscopic approach can not only achieve high negative margin, but also has better survival results than CFR in advanced Kadish C stage patients (29). In our study, compared with CFR, most patients, including with advanced tumors, obtain a higher survival rate after endoscopic surgery, which makes us believe that this is an effective and safe surgical method. This may overstate the significance of this study, because the number of patients undergoing endoscopic surgery was far more than that undergoing CFR. Direct comparison may affect the accuracy of the results. Nevertheless, based on the extensive use of endoscopic surgery in our institution without sacrificing local control, we still support that endoscopic surgery combined with radiotherapy in patients, and even patients with advanced tumors can achieve long-term survival.

Surgical margin was an important prognostic factor for survival outcomes. A recent study of 1,307 patients with malignant skull base tumors showed that the local recurrence rate doubled and the survival rate halved in patients with positive surgical margins (43). The enlarged field of vision of endoscopic surgery enables surgeons to carefully identify the tumor margin, origin and anatomical structure of the tumor. The previous study of Harvey et al. (29) proved that endoscopic surgery can obtain negative margin even in advanced tumors. Folbe et al. (44) also proved that endoscopic surgery can achieve a high rate of negative margin, even in the Kadish C stage. Nakagawa et al. (45) reported that 95.5% (21/22) of patients had negative margin after endoscopic surgery. Unfortunately, in our study, the surgical margin of patients was not accurately obtained.

Radiotherapy plays an important role in the treatment of ENB, especially in advanced tumors. It was recommended as the post-operative treatment for non-radical surgery in all T3 and T4 cases, Kadish C cases, and high-grade Hyams in our cohort. Because of the rarity of ENB, there is no uniform standard for radiation dose. We used 54–75.9 Gy for post-operative radiotherapy, 69.4–72.8 Gy in the setting of definitive radiotherapy, and the dose of pre-operative radiotherapy was unknown. Mori et al. reported a mean radiation dose of 40 Gy for pre-operative radiotherapy, 50–66 Gy range in the setting of post-operative radiotherapy, and 54–66 Gy in the setting of definitive radiotherapy (20). Yin et al. reported the radiation doses ranged from 50 to 60 Gy for pre-operative radiotherapy. The dose of post-operative radiotherapy depends on the position of surgical margin. If the surgical margin was positive, the median dose was 68 Gy (66–70 Gy); otherwise, the median dose was 66 Gy (50–70 Gy) (7). Li et al. reported that the average radiation doses for patients with pre-operative radiotherapy, post-operative radiotherapy and definitive radiotherapy were 63.53 Gy (50–71.9 Gy), 62.79 Gy (50–68.9 Gy), and 67.66 Gy (63.6–78 Gy), respectively (46). Regarding the appropriate radiation dose for ENB, further research is still needed.

**Figure 4:**
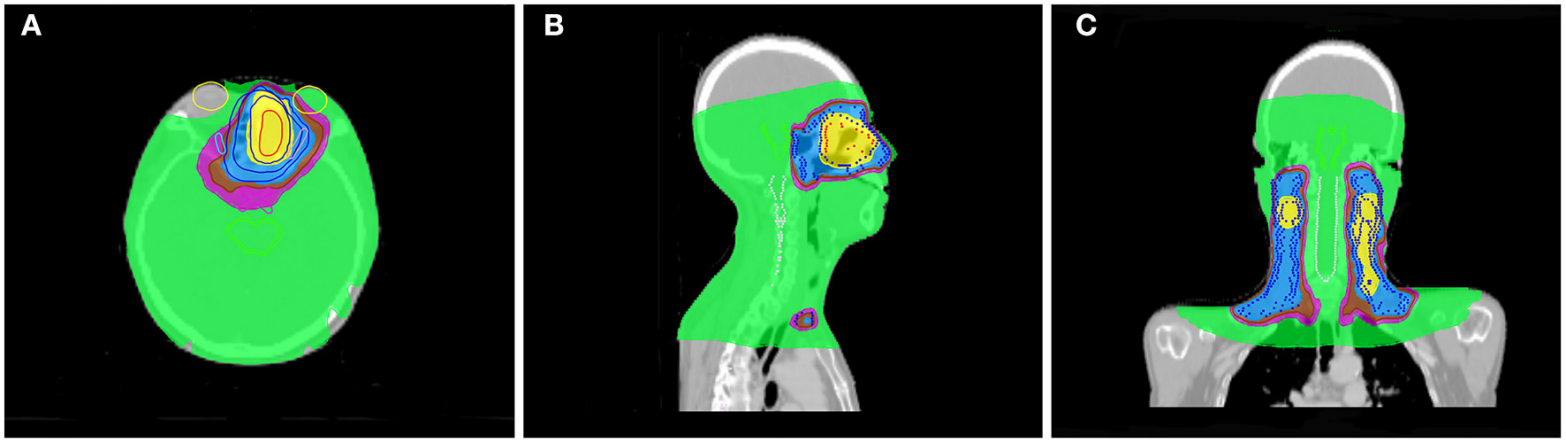
One patient distribution on Helical tomotherapy. Color-wash areas:60 Gy (yellow), 54 Gy (blue), 50 Gy (brown), 45 Gy (purple), 9 Gy (green). **(A)** refer to axial CT, **(B)** refer to sagittal CT, and **(C)** refer to coronal CT.

Radiotherapy for ENB is challenging due to adjacent radiosensitive organs such as lacrimal glands, lenses, optic nerves, and brainstem. Insufficient dose delivery due to the protection of organs at risk is a major reason for treatment failure. Modern radiotherapy technology, such as intensity-modulated radiation therapy (IMRT), that can limit the radiation dose to nearby normal tissues and do not appear to result in compromised tumor control (47). Compared with conformal radiotherapy (CRT), IMRT has better survival outcomes and less adverse reactions (48). However, IMRT has a drawback of large monitor units (MUs). Large MUs may increase the risk of secondary radiation-induced malignancies due to incremental scattered radiation and low-dose radiation to the other parts of body (49). Helical tomotherapy (HT) represents the progress of radiotherapy technology, as it shows superior results in terms of PTV coverage, and organs at risk (OAR) sparing compared to IMRT (50–52). For the protection of organs at risk, HT is particularly superior in protection of the brain stem and lens (51–53). Orbital invasion is common in ENB, and is one of the most important independent prognostic factors (28). In our cohort, 31.2% (20/64) of patients had orbital invasion at initial diagnosis. Achieving a certain dose requirement for tumor irradiation and protecting the eyes at the same time is a challenge for ENB radiotherapy. In our series, only 1 patient was treated with HT as post-operative radiotherapy, and this patient had a tumor invading the orbit at the same time. The prescription dose was 60 Gy, and the single dose was 2 Gy. Figure 4 shows the dose distribution of the patient. From the image, we can see that the irradiation of the optic chiasm, left lacrimal gland, and left lens was within the safe dose. Therefore, modern radiotherapy technology may obtain good treatment outcomes while reducing side effects.

In our study, 14 cases (21.9%) had local recurrence, 5 cases (7.8%) had cervical lymph node metastasis and 3 cases (4.7%) had distant metastasis. The basic clinical information is presented in Table 3. Local failure has been reported in nearly 10–30% of cases, while regional failure have been reported in nearly 10–20% and distant metastasis have been reported in nearly 5–10% (3, 8, 9, 17, 20, 21, 54). This suggests that patients are at relatively high risk for recurrence. It was proposed by Ow et al. that a full follow-up period of 7–10 years was necessary for patients (9). The longest time to recurrence in our study was 121 months, which is why we also advocate long-term follow-up.

Cervical lymph node metastasis is an important part of recurrence management. Most cervical lymph node metastasis occurred in level II, followed by levels I, III and retropharyngeal lymph nodes (7, 55, 56). The observations of Song et al. (54) differed somewhat from those of previous reports, as they observed that Ib and VIIa were most frequently affected after level II. In our study, lymph node metastasis most commonly occurred in level II and III. A combination of selective neck dissection and chemoradiotherapy was recommended for the treatment of cervical lymph node metastasis in our cohort. There is considerable controversy about whether patients with negative cervical lymph nodes should receive prophylactic neck radiation therapy. One study reported that elective nodal irradiation (ENI) can reduce the rate of cervical nodal failure from 10.7 to 1.6% for N0 disease, but did not improve final survival outcomes (54). Noh et al. hold a conservative attitude to the prophylactic radiation of cervical lymph nodes, and they think that the ENI can be omitted for N0 disease when patients are undergoing radiotherapy and chemotherapy (57). At our institution, no cervical nodal failure occurred in patients treated with prophylactic neck irradiation. Among the 54 patients without cervical lymph node metastasis who did not receive prophylactic neck irradiation, 5 patients (9.3%) experienced cervical lymph node metastases during the course of their disease, all of whom had Kadish C stage. The regional failure in these 5 patients during follow-up was managed via salvage treatments, and only 1 patient died 2 years after the completion of salvage treatment. The reasons why we did not support prophylactic neck irradiation were that the cervical nodal failure rate (9.3%) was low in our study and salvage treatment can save local failure and obtain good survival outcomes. Our follow-up time is long enough to make our conclusion more reliable.

**Figure 5:**
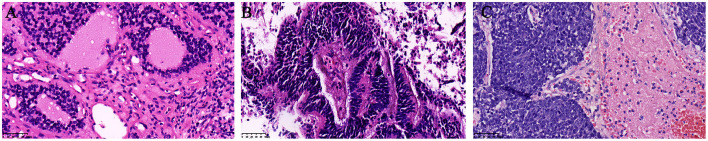
Histopathologic H&E slides of Hyams grade II, III, and IV (×400 original magnification, Hematoxylin and Eosin). **(A)** refer to Hyams grade II, **(B)** refer to Hyams grade III, and **(C)** refer to Hyams grade IV.

**Figure 6:**
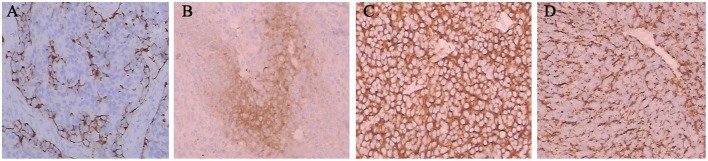
The immunohistochemical characteristics of ENB. Tumor cells were positive for S-100 **(A)**, NSE **(B)**, SYN **(C)**, and CGA **(D)**.

Prognostic factors can predict poor survival outcomes to assess whether patients need more aggressive treatment. The various prognostic factors of ENB that have been reported are age, node status, delayed nodal disease, distant metastasis, treatment modality, Hyams grade, Kadish stage, skin-involved, tumor invasion to the orbit, dural involvement, intracranial extension, and surgical margins (7, 8, 11, 14, 17, 18, 27–29, 58). And Hyams grading was the most reliable feature for predicting outcome (11). In our study, we asked professional pathologists to grade the 64 patients according to Hyams grade. However, we did not find Hyams grade I. Univariate analysis showed that Hyams grade was a significant prognostic factor of both OS and PFS. Multivariate analysis Hyams grade IV exhibited a significant poor PFS than Hyams grade I or II. According to different histological characteristics, including architecture, mitotic activity, nuclear pleomorphism, fibrillary matrix, calcification, rosette and necrosis, the Hyams grading scheme is composed of four grades (12). Grade 1 and grade 2 are characterized by neurofibrillary matrices and lobular architecture without necrosis and mitosis. Grades 3 and 4 are characterized by high mitotic activity, nuclear pleomorphism, and necrosis (Figure 5). Necrosis is common in grades 4 and occasionally in grade 3. The immunohistochemical characteristics of ENB are shown in Figure 6. Compared with Hyams low-grade ENBs, Hyams high-grade ENBs were more likely to have metastasis, and to have a lower overall survival rate (59). However, whether the Hyams grading system is better than the other 2 staging systems (Kadish stage and TNM stage) for predicting prognosis requires further study.

## Conclusions

ENB is a rare tumor. Due to the small number of patients in our retrospective study, these results provide a preliminary basis for the diagnosis and treatment of ENB. However, we have unveiled the major prognostic factors and effective treatment approaches for ENB. Surgery combined with radiotherapy with or without chemotherapy was shown to be the best treatment for ENB. Endoscopic surgical techniques may also be used as an option to provide the same or better survival outcomes for advanced patients compared with open surgery.

## Data Availability Statement

The raw data supporting the conclusions of this article will be made available by the authors, without undue reservation.

## Ethics Statement

The studies involving human participants were reviewed and approved by Medical Ethics Committee of Xiangya Hospital of Central South University (201912460). Written informed consent from the participants' legal guardian/next of kin was not required to participate in this study in accordance with the national legislation and the institutional requirements. Written informed consent was not obtained from the individual(s) for the publication of any potentially identifiable images or data included in this article.

## Author Contributions

QZ and RW: conception and design. RW and WC: administrative support. QX, LO, and MW: provision of study materials or patients. WC and YT: data collection and collation. QZ and YH: data processing and analysis. All authors: manuscript writing and final approval of manuscript.

## Conflict of Interest

The authors declare that the research was conducted in the absence of any commercial or financial relationships that could be construed as a potential conflict of interest.
